# Mosaicism for Autosomal Trisomies: A Comprehensive Analysis of 1266 Published Cases Focusing on Maternal Age and Reproductive History

**DOI:** 10.3390/genes15060778

**Published:** 2024-06-13

**Authors:** Natalia V. Kovaleva, Philip D. Cotter

**Affiliations:** 1 Academy of Molecular Medicine, Mytniskaya Str. 12/44, 191144 St. Petersburg, Russia; 2 ResearchDx, 5 Mason Ln, Irvine, CA 92618, USA; pcotter@researchdx.com

**Keywords:** mosaic autosomal trisomies, prenatal diagnosis, true fetal mosaicism, confined placental mosaicism, intrauterine growth restriction, fetal losses, miscarriages, uniparental disomy, advanced maternal age, maternal reproductive history

## Abstract

Mosaicism for autosomal trisomy is uncommon in clinical practice. However, despite its rarity among both prenatally and postnatally diagnoses, there are a large number of characterized and published cases. Surprisingly, in contrast to regular trisomies, no attempts at systematic analyses of mosaic carriers’ demographics were undertaken. This is the first study aimed to address this gap. For that, we have screened more than eight hundred publications on mosaic trisomies, reviewing data including gender and clinical status of mosaic carriers, maternal age and reproductive history. In total, 596 publications were eligible for analysis, containing data on 948 prenatal diagnoses, including true fetal mosaicism (TFM) and confined placental mosaicism (CPM), and on 318 cases of postnatally detected mosaicism (PNM). No difference was found in maternal age between normal pregnancy outcomes with appropriate birth weight and those with intrauterine growth restriction. Unexpectedly, a higher proportion of advanced maternal ages (AMA) was found in normal outcomes compared to abnormal ones (abnormal fetus or newborn) and fetal losses, 73% vs. 56% and 50%, *p* = 0.0015 and *p =* 0.0011, correspondingly. Another intriguing finding was a higher AMA proportion in mosaic carriers with concomitant uniparental disomy (UPD) for chromosomes 7, 14, 15, and 16 compared to carriers with biparental disomy (BPD) (72% vs. 58%, 92% vs. 55%, 87% vs. 78%, and 65% vs. 24%, correspondingly); overall figures were 78% vs. 48%, *p =* 0.0026. Analysis of reproductive histories showed a very poor reporting but almost two-fold higher rate of mothers reporting a previous fetal loss from PNM cohort (in which almost all patients were clinically abnormal) compared to mothers from the TFM and CPM cohorts (with a large proportion of normal outcomes), 30% vs. 16%, *p* = 0.0072. The occurrence of a previous pregnancy with a chromosome abnormality was 1 in 13 in the prenatal cohort and 1 in 16 in the postnatal cohort, which are five-fold higher compared to published studies on non-mosaic trisomies. We consider the data obtained in this study to be preliminary despite the magnitude of the literature reviewed since reporting of detailed data was mostly poor, and therefore, the studied cohorts do not represent “big data”. Nevertheless, the information obtained is useful both for clinical genetic counseling and for modeling further studies.

## 1. Introduction

Non-mosaic chromosomal trisomies are a common cause of fetal loss and congenital malformations in liveborn carriers. The only viable non-mosaic trisomies involved chromosomes 21, 18, and 13. In a recent review, Benn and Grati [1] highlighted that these three chromosomes, in order, carry the least amount of protein-coding genes. Trisomies involving other chromosomes are lethal, and among them, there is a large excess of trisomy 16 cases (25% of all trisomies) despite an approximately average number of protein-coding genes. These authors suggest that each specific abnormality might have its own risk for embryonic arrest or failure to implant, spontaneous abortion, or abnormal live birth [1].

Various parameters of parental demographics as the cause of chromosomal abnormalities (predominantly of trisomy 21, the most frequent numeric chromosomal abnormality) were the subject of intensive studies for many decades, including grandmaternal age [2,3], paternal age [4,5], consanguinity [6,7], ethnic variations [8,9], socioeconomic status [10,11], as well as other potential risk factors, e.g., reproductive behavior, habitual and environmental factors, and occupational exposures. Finally, the most convincing factor, apart from gonadal mosaicism, appeared to be maternal advanced age. Poor reproductive history, namely history of miscarriages, was also found to be a factor increasing the risk of abnormal offspring with chromosomal abnormality [12,13]

Mosaic trisomies are infrequently diagnosed postnatally, comprising a minority of the clinical cytogenetic diagnoses, probably greater than 1 in 10,000 [14], varying depending on the involved chromosomes and diagnostic policies/limitations in the testing laboratory (examined tissue(s), number of examined cells, and availability of modern technologies). Moreover, in contrast to carriers of non-mosaic trisomy, a proportion of mosaic carriers can go undiagnosed for extended periods because of the absence of dysmorphic features being, for example, educationally subnormal [15]. Carriers of gonadal mosaicism might not be diagnosed until the delivery of an abnormal child or a recurrent miscarriage. Prenatally diagnosed cases of mosaicism account for less than one percent of tested cases [16]. For example, among 15,362 tested pregnancies, only one was confirmed to be TFM for trisomy 7, which is one of the most common mosaic findings in prenatal diagnoses [17].

The mechanisms of mosaicism formation are more diverse than those of non-mosaic trisomies [18]. In addition to postzygotic origin, mosaicism for regular trisomies may arise due to meiotic nondisjunction followed by mitotic loss of the trisomic chromosome, also known as “trisomy correction” or “trisomy rescue”. This mechanism leads to the restoration of biparental inheritance (BPD, biparental disomy) in two-thirds of the cases and uniparental disomy (UPD) in one-third of the cases. This may lead to the expression of clinical features of both mosaic trisomy and UPD. Few cases of mixed mosaic trisomy in the same individual have been described, where a cell line with BPD coexists with a cell line with UPD [19]. UPD-associated disorders occur if the chromosomes involved contain differentially methylated regions (DMRs), which may express differently depending on the parental origin [20], for example, relatively mild Temple syndrome (maternal UPD(14) in contrast to lethal Kagami–Ogata syndrome (paternal UPD(14)

Therefore, demographic data obtained from studies on cohorts of non-mosaic trisomies might not be applicable or misleading when considering cases of mosaic trisomies. Since, according to our best knowledge, no attempts at systematic analysis of mosaic carriers’ demographics were undertaken, we were determined to review the literature. The goals of this project were (i) a study of mosaic profile and clinical status in cohorts of carriers of true fetal mosaicism (TFM) and confined placental mosaicism (CPM); (ii) comparison of mosaicism profiles in prenatally diagnosed carriers (both TFM and CPM) with carriers of postnatally diagnosed mosaicism (PNM); (iii) a comparative study on maternal ages, reproductive histories and pregnancy outcomes in prenatally detected carriers vs. carriers of postnatally diagnosed mosaicism.

## 2. Material and Method

The data for this study were obtained from literature identified from various sources, including PubMed, Research Gate, and ChromosOmics UPD Database [19]. We screened more than eight hundred publications on mosaic trisomies for the presence of the data of interest, including gender and clinical status of mosaic carriers, maternal age and reproductive history. In total, 596 publications containing data on 948 prenatal diagnoses and 318 postnatal diagnoses were selected for the analysis. Maternal age was reported in 546/948 (58%) prenatally diagnosed cases and 217/318 (68%) postnatally diagnosed cases. Pregnancy outcome was indicated in 796/948 (83%) cases, maternal reproductive history was reported in 339/1272 (27%) cases, and parental origin of the euploid line was determined in 179 cases.

Prenatal diagnoses were divided into true fetal mosaics (TFM) and mosaics confined to placenta (CPM). According to common practice, mosaicism detected in either direct chorionic villi samples (CVS), in cultured CVS, or in both, but not in amniocytes, was classified as confined placental mosaicism [21,22]. According to another diagnostic approach, where no villus samples were tested, amniocentesis was performed instead. If mosaicism detected in amniocytes was not confirmed in fetal cord blood, CPM was concluded [23,24]. Those cases reported to have no confirmatory study (most frequently because of elective termination of pregnancy or miscarriage) or for which data were not available fall into the third category—Not confirmed.

Pregnancy outcomes were classified as abnormal when a fetus or newborn had a structural abnormality or multiple abnormalities, as well as dysmorphic features, developmental delay, mental retardation at postnatal follow-up; isolated intrauterine growth restriction (IUGR) and isolated pigmentary abnormality were not considered abnormal. Cases with clinical manifestation of UPD were excluded when comparing normal vs. abnormal outcomes since their abnormality did not involve a concomitant mosaicism in most cases.

Maternal reproductive history was classified as uncomplicated in two instances: (i) when pregnancy numbers were concordant with the number of outcomes, including the examined pregnancy/patient, and (ii) when the absence of spontaneous abortions/miscarriages was clearly stated. Reproductive history was considered complicated when any abnormal conditions or previous abnormal outcomes were reported. Spontaneous abortions, missed abortions, intrauterine fetal birth, and stillborn were classified as fetal loss. Infertility, previous fetus/child with chromosomal abnormality or with congenital malformations, ectopic pregnancy, hydatidiform mole, polycystic ovarian syndrome, ovarian cystic teratoma, and pregnancy termination for unspecified reason were classified as Other complications. Cases of discordance between the number of pregnancies and the number of outcomes (for example, G8P3 or multigravida nulliparous) were classified as Inconclusive reports.

Statistical analysis was performed using software LePAC (https://eris62.eu/ErisLePAC.html, accessed on 11 June 2024) for an estimation of 95% confidence intervals (CI) for proportions, their differences and ratios; StatXact (https://www.cytel.com/software/statxact/, accessed on 11 June 2024) for exact point and interval estimation of the parameters of multinomial distribution as well as the Fisher–Freeman–Halton test for contingency tables RxC; MOVER-D (https://profrobertnewcomberesources.yolasite.com/, accessed on 11 June 2024), which calculates a confidence interval for a difference of two quantities, starting from independent estimates and confidence intervals for both; Fisher’s exact test *p*-value calculator, 2 × 2 and 2 × 3 (https://www.cog-genomics.org/software/stats, accessed on 11 June 2024), which calculates the mid-*p*-values for the Fisher’s exact tests.

## 3. Results and Discussion

### 3.1. Mosaic Trisomy Profile and Clinical Outcomes in Prenatal Diagnoses. Comparing Mosaic Trisomy Profile in Prenatal and Postnatal Diagnoses

From the 596 selected papers, we retrieved data on 1266 cases of mosaic trisomies for chromosomes 1–22 (mosT1–mosT22). Despite every effort to collect as many cases of mosaics for rare trisomy as possible, only a few cases involving chromosomes 1, 4, 11 and 19 were identified. TFM was diagnosed in 271 cases, CPM in 553 cases, Not confirmed in 130 cases, and PNM in 317 cases. The data are presented in Table 1 and Table 2.

In prenatal diagnoses, the involvement of different chromosomes in every studied category is disproportional, varying from 0 (mosT1 and mosT19) to 28 cases (mosT9) in the TFM cohort and to 64 (mosT7) and 65 (mosT16) cases in the CPM cohort. The rate of some chromosomes might be different between TFM and CPM, for example, 6% vs. 12% of mosT7 and 7% vs. 1% of mosT16, but no statistically significant difference was found. However, we should compare the chromosome involvement with caution, since the mode of the data collection in this study might not allow for completely unbiased samples.

Expectedly, in the CPM cohort, normal outcomes prevailed compared to abnormal. However, the most apparent differences between the TFM and CPM cohorts were not the overall proportion of normal (34% vs. 67%) and abnormal outcomes (52% vs. 13%) but the chromosome-specific relations between normal and abnormal outcomes. More abnormal than normal (including or not including IUGR) outcomes were reported for mosaic trisomies 2, 4, 5, 9, 14–18, and 22. In contrast, more normal (including or not including IUGR) than abnormal outcomes were reported for mosaic trisomies 7, 8, 12, 13, and 21. The prevalence of IUGR did not differ significantly, i.e., 10% vs. 15%, while the proportion of fetal losses was 5% in both. It should be noted that in these groups, TFM (generalized mosaicism) and CPM (mosaic placental and a fetus with an apparently normal chromosome set), the reasons for fetal loss were likely different.

The cohort of non-confirmed cases demonstrated an intermediate rate of abnormal outcomes, being a mixture of TFM and CPM cases. A higher rate of fetal losses is explained by the accumulation of cases not followed up because of various reasons, including a poor growth of cells from a product of conception. In this group, we see a higher number of mosaic trisomies 9 and 14 among abnormal outcomes, while all other mosaic trisomies prevail among normal outcomes, likely indicating a predominantly CPM origin.

Since no statistically significant difference among the TFM and CPM cohorts was observed, all three prenatal cohorts were pooled and compared with the postnatal cohort (Table 2). 

A comparison of the data collected from different sources should be performed with caution because of the highly probable publication bias in favor of most rare trisomies and underreporting of “less interesting” common mosaics. This may be the reason for the mosT14 prevalence over mosT21 in postnatal diagnoses (n = 27 vs. n = 40), in contrast to their numbers in prenatal diagnoses (n = 22 vs. n = 61). Nevertheless, it is remarkable that some mosaic trisomies found in appreciable numbers in prenatal diagnoses are exceptionally infrequent (mosT4 and mosT6) or absent (mosT5 and mosT11) in postnatal diagnoses. At the same time, among prenatally detected cases of the listed mosaic trisomies, there were 4, 13, 9, and 8 normal outcomes, which might explain their rarity among postnatal (mostly abnormal) diagnoses.

### 3.2. Maternal Age Distribution in Prenatal Diagnoses, Normal Outcomes vs. Abnormal

Since offspring with IUGR represents an appreciable part of outcomes, we performed a preliminary comparative study, which showed no difference in the proportion of mothers with advanced maternal ages (AMA, ≥35 yr) between normal pregnancy outcomes with appropriate birth weight (n = 234) and those with intrauterine growth restriction but otherwise normal (n = 48), 74% and 73%, correspondingly Table 3).

Data on maternal age distributions are presented in Table 4. No statistically significant difference was found between TFM and CPM in both cohorts, so they were pooled, and those cases that were not precisely identified as TFM or CPM due to termination of pregnancy or because of lack of information on follow-up were added. Overall, in contrast to reasonable expectation, mothers of normal offspring appeared to be older compared to those with abnormal pregnancy outcomes (average age of 35.8 yr vs. 33.9 yr). The proportions of AMA mothers were 73% vs. 56%, correspondingly, *p* = 0.0015. One may suggest an enhanced selective miscarriage of abnormal pregnancies with advancing maternal age, as it was demonstrated for trisomy 21 pregnancies [25], as well as for other trisomies [26]. However, women with pregnancy loss were younger than mothers of normal offspring and slightly younger than those with abnormal outcomes, with an average age of 33.3 yr and 50% proportion of AMA. *p =* 0.0011.

### 3.3. Maternal Age and Parental Origin of Diploid Cell Line

Recent observation of a higher proportion of older mothers in mosaic carriers with concomitant UPD(14) [27] prompted us to verify this phenomenon on a larger sample of mosaic trisomies. We have identified cases with the largest numbers of known parental origin of diploid cell line and known maternal age. These were mosT7 (n = 14), mosT14 (n = 23), mosT15 (n = 24), and mosT16 (n = 34) (Table 5).

In all these groups, a higher AMA proportion was found for carriers of concomitant UPD compared to carriers with BPD (72% vs. 58%, 92% vs. 55%, 87% vs. 78%, and 65% vs. 24%, correspondingly). Overall figures were 78% vs. 48%, *p =* 0.0026. These data cannot be explained by random unequal distribution into prenatal and postnatal cohorts since UPD and BPD cases appeared to be well balanced regarding their numbers and their sources.

This is a quite intriguing finding because of the common mechanism of both UPD and BPD formation, i.e., the correction of original trisomy by loss of an extra chromosome. If this phenomenon is confirmed in future studies, one may speculate on a special mechanism of preferential disposal of the chromosome inherited from the other parent, associated with advancing maternal age.

### 3.4. Maternal Reproductive History in Prenatal Diagnoses vs. Postnatally Diagnosed Mosaicism

Analysis of published cases revealed the absence or poor reporting of maternal reproductive history in the majority of them. Only 339 histories were available for the analysis (Table 6).

Among them, four categories were informative: primigravidas, complicated histories, uncomplicated histories, and fetal loss. The category ‘other complications’ included histories of pregnancy termination with no reason indicated. Some of them may be pregnancy loss. There is the same problem with the category ‘inconclusive reports’, containing cases with a discrepancy between the number of pregnancies and the number of deliveries.

Nevertheless, the data collected allowed justifying the comparison and subsequent pooling of the TFM and CPM cohorts against postnatal cohort. The overall frequency of mothers having had previous fetal loss(es) in the prenatal cohort was 16% vs. 30% in the postnatal cohort, *p* = 0.0072. Advancing maternal age is associated with enhancing miscarriage [28,29]. However, mothers from the prenatal cohort reporting previous miscarriage(s) and those with uncomplicated reproductive history did not differ by a proportion of AMA, 67% and 66%, respectively. In the postnatal cohort, corresponding figures were _25_41_59_% and _16_30_47_%; therefore, the expected difference was not statistically significant due to small samples.

In the ‘other complications’ cohort, there were 10 mothers of fetuses reported with various mosaic trisomies, having had a previous offspring with a different chromosomal abnormality, including eight cases with T21, which may be interpreted as a genetic predisposition to chromosome nondisjunction. In addition, there were four cases of trisomy 21 recurrence, suggestive of parental gonadal mosaicism. The occurrence of a previous chromosome abnormality in the prenatal cohort, seems to be extremely high, 1 in 13 (14/180 excluding 57 primigravidas) or _4.7_.8.1_13.0_%. As a history of chromosomal anomaly is one of the most common indications for prenatal testing, we compared this figure with published data on the occurrence of autosomal trisomies in prenatal testing for previous de novo chromosome abnormality. Among 2,623 diagnoses for this indication, there were 37 cases of abnormal fetus, i.e., 1 in 79 or _1.0_1.4_1.9_% [30]. Caron et al. reported a similar figure of 1 in 72 (17/1232) or _0.91_.4_2.2_%, while among 2490 diagnoses for AMA indication, previous occurrence of autosome trisomies was 1 in 102 (247/24,901) [31]. Differences between the data from this study and published data are highly significant, with *p* = 2^.^× 10^−6^ nd *p* = 7 × 10^−6^, correspondingly. The reason for this difference between mosaic and nonmosaic aneuploidies is unclear.

In the postnatal cohort, six cases were identified with proven or suggested parental gonadal mosaicism: a woman with hysteria and her daughter suffered from undiagnosed mental illness, both with mosT7 [32]; a boy and his mother, both with mosT10 [33]; a mother and her two daughters with mosT13 [34]; a girl with mosT21 and her mother reported with three previous spontaneous abortions [35]; two non-twin siblings with mosT18 [36]; and two non-twin siblings with mosT21 [37]. Among 98 postnatal cases with known maternal reproductive history, the occurrence of “transmitted” mosaicism was 1 in 16, which is very similar to the finding in the prenatal cohort.

The inheritance of chromosomal mosaicism is not uncommon; for example, mosT21 in successive generations was documented in 12 of 80 families of carriers of gonadal mosaicism [38]. It was reported repeatedly that gonadal mosaicism may account for more cases than it detected cytogenetically in somatic tissue(s) [39,40,41,42]. At the same time, theoretically, a large proportion of carriers of gonadal mosaicism can be identified by analyzing the proband’s trisomic line for the presence of two homologues from the same grandparent [43]. However, no subsequent reports on uncovering parental gonadal mosaicism using this approach were published.

## 4. Conclusions

This study reports non-trivial maternal age correlations with some clinical parameters of the mosaic offspring and with the mechanism of mosaicism formation. Regarding mosaic profiles, especially in the postnatal cohort, we reiterate that a limitation of the study is the data sources, particularly a potential publishing bias towards rare cases and underreporting of less interesting mosaics for common trisomies. Another problem is the very poor reporting of demographic data, including gender of mosaic carriers, maternal age and reproductive history. We suggest that further investigations should be based either on national registers or on international collaboration, which will strengthen (or disprove) the findings reported here.

## Figures and Tables

**Table 1:** Number of prenatally diagnosed cases by chromosome.

Chromosome	True Fetal Mosaicism						Confined Placental Mosaicism						Not Confirmed or Data Unavailable					
	Normal	IUGR	Abnormal	Fetal Loss	Not Specified	Total	Normal	IUGR	Abnormal	Fetal Loss	Not Specified	Total	Normal	IUGR	Abnormal	Fetal Loss	Not Specified	Total
1	0	0	0	0	0	0	0	0	0	0	0	0	0	0	0	0	0	0
2	2	2	9	1	0	14	13	6	7	1 ^a^	2	29	8	0	2	5	2	17
3	1	2	1	0	4	8	18	2	1	1	2	24	4	0	0	0	3	7
4	1	0	6	0	0	7	0	0	0	0	0	0	3	0	0	1	0	4
5	2	0	5	0	0	7	7	3	1	0	2	13	1	0	0	0	0	1
6	7	0	5	1	0	13	4	3	0	0	0	7	1	0	0	0	0	1
7	3	2	6	1	3	15	36	4	3	3	18	64	7	0	0	0	7	14
8	12	0	5	1	4	22	16	10	0	0	10	36	4	0	1	0	0	5
9	6	2	19	0	1	28	14	2	3	2	7	28	2	0	8	2	0	12
10	3	0	0	0	0	3	7	0	2	0	2	11	0	0	0	1 ^a^	1	2
11	1	0	1	1	0	3	6	0	0	0	0	6	1	0	0	0	1	2
12	12	7	0	0	0	19	25	1	4	0	3	33	3	0	1	2	0	6
13	7	1	5	2	3	18	21	3	1	3	5	33	1	0	0	1	1	3
14	1	0	4	0	2	7	4	3	0	0	2	9	1	0	4	1	0	6
15	2	0	7	0	1	10	14	1	6	2	4	27	2	0	0	0	2	4
16	1	2	14	1	1	19	16	16	15	6	12	65	3	0	1	3 ^a^	0	7
17	1	1	9	0	0	11	19	1	1	0	0	21	6	0	1	1 ^a^	0	8
18	3	1	8	2	5	19	30	4	1	1	10	46	4	0	1	3 ^a^	1	9
19	0	0	0	0	0	0	0	0	0	0	0	0	0	0	0	0	1	1
20	7	0	8	0	0	15	30	0	4	1	7	42	5	0	0	0	2	7
21	8	1	4	0	6	19	15	3	3	2	9	32	1	0	0	1	8	10
22	1	2 ^c^	10	2 ^b^	0	15	7	5	7	2	5	26	2	0	0	2	0	4
Total	81	23	126	12	30	272	302	67	59	24	100	552	59	0	19	23	29	130
Proportion, excluding Not specified	33%	10%	52%	5%		n = 242	67%	15%	13%	5%		n = 452	58%	0%	19%	23%		n = 102

^a^ Including one case of fetal anomaly; ^b^ including two cases of fetal anomaly; ^c^ including one case of neonatal death.

**Table 2:** Mosaic trisomies profiles in prenatal and postnatal diagnoses.

Chromosome	1	2	3	4	5	6	7	8	9	10	11	12	13	14	15	16	17	18	19	20	21	22
Prenatal diagnoses, n = 948	0	60	35	11	21	15	93	64	68	16	11	58	55	22	41	91	40	74	1	65	63	44
Postnatal diagnoses, n = 318	2	6	8	1	0	1	15	30	31	7	0	20	35	40	10	6	5	32	1	10	27	31

**Table 3:** Comparison of maternal age in newborns with weight appropriate to gestation age (normal birth weight) and with intrauterine growth restriction.

Maternal Age, yr	Normal Birth Weight	Intrauterine Growth Restriction	Total
<20	1	1	2
20–24	5	2	7
25–29	13	5	18
30–34	42	5	47
35–39	112	20	132
40–44	41	14	55
45+	1	1	2
AMA	19	0	19
Total	234	48	282
Proportion of AMA	74%	73%	74%

**Table 4:** Maternal age and pregnancy outcome.

Maternal Age, yr	Normal Outcome				Abnormal Outcome				Prenatal Loss
	TFM	CPM	Not Confirmed	Total	TFM	CPM	Not Confirmed	Total	
<20	0	2	0	2	4	0	0	4	0
20–24	2	3	1	6	4	5	1	10	2
25–29	5	12	1	18	16	5	2	23	5
30–34	20	28	0	48	23	10	3	36	10
35–39	31	91	7	129	41	13	4	58	11
40–44	22	24	9	55	17	8	0	25	2
45+	0	1	0	1	2	1	0	3	
AMA	2	13	0	15	1	3	0	4	4
Total	82	174	18	274	108	45	10	163	34
AMA proportion	73%				56% *				50% **
Mean age, yr	35.8				33.9				33.3

* Difference between 73% and 56% statistically significant at *p =* 0.0015. ** Difference between 73% and 50% statistically significant at *p* = 0.0011.

**Table 5:** Parental origin of diploid cell line in mosaic trisomies 7, 14, 15, and 16 according to maternal age.

Maternal Age, yr	Uniparental Disomy					Biparental Disomy				
	7	14	15	16	Total	7	14	15	16	Total
<20	0	0	0	0	0	1	1	1	0	3
20–24	0	0	0	0	0	2	1	1	0	4
25–29	1	0	0	4	5	0	2	0	5	7
30–34	1	1	2	2	6	0	1	0	8	9
35–39	2	5	4	10	21	3	2	6	1	12
40–44	3	6	5	1	15	1	4	1	3	9
45+	0	0	2	0	2	0	0	0	0	0
AMA	0	0	2	0	2	0	0	0	0	0
Total	7	12	15	17	51	7	11	9	17	44
Proportion of AMA	72%	92%	87%	65%	78%	58%	55%	78%	24%	48% *
Mean age, yr	37.3	37.9	40.3	33.9	37.2	33.3	33.8	34.1	32.5	34.2

* Difference between 78% and 48% is significant at *p =* 0.0026.

**Table 6:** Maternal age and reproductive history profiles in cohorts of prenatally and postnatally diagnosed carriers of mosaicism.

Maternal Age, yr	True Fetal Mosaicism						Confined Placental Mosaicism						Postnatal Diagnoses					
	Primigravidas	Uncomplicated History	Previous Fetal Loss	Other Complications	Inconclusive Reports	Total	Primigravidas	Uncomplicated History	Previous Fetal Loss	Other Complications	Inconclusive Reports	Total	Primigravidas	Uncomplicated History	Previous Fetal Loss	Other Complications	Inconclusive Reports	Total
<20	4	0	0	0	0	4	0	1	0	0	0	1	0	0	0	1	0	1
20–24	3	1	0	2	0	6	3	2	0	1	0	6	5	5	2	2	1	16
25–29	7	7	1	4	1	20	5	2	2	3	0	12	6	9	10	4	1	30
30–34	6	6	5	8	9	34	8	2	4	2	3	19	5	8	4	5	0	22
35–39	11	15	7	10	6	49	7	14	9	10	2	42	2	8	5	2	0	17
≥40	1	5	6	5	5	22	1	6	0	6	2	15	0	1	6	2	2	11
AMA	0	0	1	0	0	1	0	0	1	0	0	1	1	0	0	0	0	1
Not stated	0	0	0	0	1	1	1	0	2	1	0	4	1	0	3	0	0	4
Total	32	34	20	29	22	137	25	27	18	23	7	100	20	31	30	16	4	102
Proportion	23%	25%	15%	21%	16%		25%	27%	18%	23%	7%		20%	31%	30% *	16%	4%	
AMA proportion	53%						60%						31%					

* Difference with average figure of 16% in prenatal cohorts is statistically significant, *p* = 0.0072.

## Data Availability

The data on the cases collected for this study along with the reference list will be available upon an appropriate request.
